# Perceived AI acceptance and occupational well-being among university physical education teachers: a cross-sectional serial mediation model of digital well-being and innovative work behavior

**DOI:** 10.3389/fpsyg.2026.1999813

**Published:** 2026-10-26

**Authors:** Jun Miao

**Affiliations:** Basic Teaching Department, Sichuan Film and Television University, Chengdu, China

**Keywords:** chain mediating effect, digital well-being, innovative work behavior, occupational well-being, perceived AI acceptance, teacher professional development

## Abstract

**Objective:**

This study aimed to test a serial mediation model examining whether perceived AI acceptance is indirectly associated with university physical education (PE) teachers’ occupational well-being through digital well-being and innovative work behavior, as proposed on the basis of Conservation of Resources theory.

**Methods:**

A cross-sectional design was adopted, and 807 university PE teachers were recruited from multiple universities in six provinces and municipalities of China through online convenience and snowball sampling. Self-report scales were employed to measure perceived AI acceptance (adapted from the Technology Acceptance Model), digital well-being (a researcher-developed instrument based on a published theoretical framework), innovative work behavior (general innovative behavior in one’s work role), and occupational well-being (the Job-related Affective Well-being Scale). The measurement structure was examined by confirmatory factor analysis, and indirect associations were estimated by bootstrapping (PROCESS Model 6, 5,000 resamples) using observed composite scores.

**Results:**

Perceived AI acceptance was significantly and positively associated with occupational well-being (total effect *B* = 0.181, 95% CI [0.143, 0.220]). Three indirect associations were observed: via digital well-being (*B* = 0.042, 95% CI [0.027, 0.059]), via innovative work behavior (*B* = 0.040, 95% CI [0.026, 0.056]), and via digital well-being and innovative work behavior in sequence (*B* = 0.021, 95% CI [0.015, 0.030]). The total indirect effect was 0.103, accounting for 56.91% of the total effect, while the direct effect accounted for 43.09%. Bootstrap confidence intervals for all indirect effects excluded zero.

**Conclusion:**

Because the data are cross-sectional and self-reported, the results indicate statistical indirect associations and not temporal or causal mediation. The observed pattern is consistent with a Conservation of Resources-based sequence from perceived AI acceptance to digital well-being, innovative work behavior, and occupational well-being, which is theoretically proposed and not empirically established. The findings suggest that digital well-being and supportive conditions for innovative work behavior may merit attention in efforts to support PE teachers’ occupational well-being during AI integration; longitudinal research is needed to test these possibilities.

## 1 Introduction

Occupational well-being (OW), broadly defined as the pattern of pleasurable and activated affective experiences that individuals derive from their work role, is a core indicator of employees’ psychological adaptation to their occupational environment (Warr, 1990; Van Katwyk et al., 2000). As a workforce whose professional practice continuously mobilizes cognitive, emotional, and physical resources, teachers’ occupational well-being has been closely linked to instructional quality, professional retention, and the broader sustainability of the educational workforce (Warr, 1990; Wang et al., 2025). Among teacher subgroups, university physical education (PE) teachers occupy a distinctive occupational niche: their work combines embodied, face-to-face instruction with competitive sport supervision, research productivity expectations, and—increasingly—digital and intelligent teaching tools, a combination that renders their occupational well-being particularly sensitive to ongoing technological change (Cui et al., 2025; Yi et al., 2025). Because PE teachers’ occupational well-being is closely intertwined with the quality of embodied instruction and student engagement in physical activity, understanding how AI-related technological appraisal is associated with this well-being is not only an occupational question but also a core educational-psychology concern regarding how teachers, as central agents of the teaching-learning process, adapt to a changing pedagogical environment.

The theoretical relevance of this population goes beyond convenience. Compared with teachers whose work is largely screen-based or text-based, PE teachers’ core instructional practice is embodied and demonstrated in real time through physical movement, so AI tools (e.g., movement analysis, training-load monitoring, automated feedback) are less likely to be the default medium of teaching and more likely to be layered on top of it as an additional demand. The appraisal of AI’s usefulness and ease of use (acceptance) may therefore bear more directly on the balance between resource investment and resource loss than in disciplines in which digital tools are already the default medium. In addition, PE teachers typically combine instruction with training, competition supervision, and research obligations, and their professional identity is often anchored in embodied expertise rather than digital competence (Yi et al., 2025; Yang et al., 2026), which may shape how they regulate digital demands (digital well-being) and how they translate AI-related appraisals into discretionary innovative behavior. We therefore regard university PE teachers as a theoretically informative case in which the resource implications of AI acceptance are likely to be salient, and not merely as a convenient sample. At the same time, the present study did not include a comparison group of other university teachers, so whether these processes differ from those of other teachers remains an empirical question.

The rapid diffusion of artificial intelligence (AI) into higher education has repositioned technology acceptance as a salient occupational variable. Perceived AI acceptance, commonly operationalized through Davis’s Technology Acceptance Model (TAM) as perceived usefulness (PU) and perceived ease of use (PEOU), captures the extent to which individuals appraise AI-based instructional and administrative tools as beneficial and effortless to use (Davis, 1989; Zhang et al., 2023). In the present study, perceived AI acceptance denotes a teacher’s cognitive appraisal of AI tools’ usefulness and ease of use; it is distinct from behavioral intention to use AI and from actual AI use, neither of which was measured. Extensive evidence confirms that PU and PEOU are robust predictors of AI adoption intention across educational contexts (Venkatesh and Bala, 2008; Zhang et al., 2023), yet comparatively little attention has been paid to whether, and through what mechanisms, such technological appraisal is associated with teachers’ broader occupational well-being—particularly for PE teachers, whose professional identity has historically been anchored in embodied pedagogy rather than digital mediation (Yi et al., 2025; Yang et al., 2026).

Existing research addressing AI and employee or teacher well-being has largely converged on two separate mechanisms that have rarely been examined jointly. A first, ‘digital resource’ pathway emphasizes that effective digital tool use is associated with individuals’ capacity to regulate information load, attention, and connectivity—that is, their digital well-being—which in turn is linked to broader well-being outcomes (Han X. et al., 2025). A second, ‘innovation’ pathway emphasizes that AI, when appraised as a usable and useful job resource, is associated with employees’ innovative work behavior (IWB), the intentional generation, promotion, and realization of novel ideas (Janssen, 2000), which is itself linked to well-being, an association often attributed to enhanced competence and engagement (Scott and Bruce, 1994; Teng et al., 2026). However, these two strands have largely been examined separately, which leaves a specific question unanswered by existing AI-acceptance and teacher well-being studies: whether the association between AI acceptance and occupational well-being is shared with an ordered combination of a personal-condition resource (digital well-being) and resource-investing behavior (innovative work behavior), or whether each is associated with this link independently. AI-acceptance studies typically stop at adoption intention or use, whereas teacher well-being studies typically examine digital strain or innovation in isolation; neither can indicate how much of the acceptance-well-being association is statistically shared with digital well-being, with innovative work behavior, and with the two in sequence. A serial model can separate these three indirect associations and thereby adds explanatory value that single-mediator models cannot. Because the data are cross-sectional, this contribution lies in testing whether the observed associations are consistent with a theoretically proposed sequence, and not in demonstrating a temporal or causal process.

Accordingly, drawing on Conservation of Resources (COR) theory (Hobfoll, 1989; Hobfoll et al., 2018), which posits that resource gains tend to unfold as caravans or spirals in which one resource is accompanied by the acquisition of further resources, the present study proposes a serial mediation model. Rather than labeling every construct generically as a “resource,” we assign each construct a distinct conceptual role. Perceived AI acceptance is treated as a cognitive appraisal of whether a new technology is likely to add to or drain personal resources, and not as a resource in itself. Digital well-being is treated as a personal-condition resource: a state of digital skills and low digital strain that indicates how much regulatory capacity teachers have left for other demands. Innovative work behavior is treated as discretionary resource investment, because generating, promoting, and realizing new ideas requires time and effort that, in COR terms, individuals are more likely to commit when they have resources to spare and expect returns. Occupational well-being is treated as an affective outcome that is consistent with resource gain (Hobfoll, 1989; Halbesleben et al., 2014; Hobfoll et al., 2018). The order of these constructs is a theoretical proposition about how this COR logic might be arranged, and is not a temporal sequence demonstrated by the present data; the reverse arrangement is also plausible and is acknowledged in Section 2.4. By adopting COR theory as a conceptual lens, this study aims to examine whether the associations observed among these four variables are consistent with the proposed COR-based sequence among university PE teachers in an increasingly digitalized higher-education context.

## 2 Literature review and hypothesis development

Note on inference. All hypotheses below are examined with data on four constructs measured at the same time point. The terms “mediation,” “mediating,” and “serial pathway” are therefore used in a statistical sense only, to denote indirect associations estimated in a regression framework. Significant indirect effects can support a statistical mediation model but cannot establish that digital well-being or innovative work behavior actually transmit the effect of perceived AI acceptance over time. Likewise, directional wording in the hypotheses reflects their theoretical framing and not a demonstrated temporal or causal order.

### 2.1 Perceived AI acceptance and occupational well-being

Perceived AI acceptance has become a pervasive occupational appraisal in contemporary higher education, exerting broad associations with faculty members’ professional practice and psychological experience. Rooted in TAM, perceived AI acceptance refers to the extent to which an individual believes that using AI-based tools will enhance job performance (perceived usefulness) and can be operated with minimal effort (perceived ease of use) (Davis, 1989; Venkatesh et al., 2003). Although perceived usefulness and ease of use are theoretically distinct beliefs in the original TAM, they are treated here as two dimensions of a single higher-order appraisal, because the present study is concerned with teachers’ overall evaluation of AI tools as beneficial and easy to use and not with the separate effects of the two beliefs; in TAM research the two are strongly interrelated, with ease of use shaping perceived usefulness, and the two indicators loaded satisfactorily on one latent factor in the present CFA (Section 4.3). The construct reflects a perceptual appraisal of AI tools and is distinct from behavioral intention to use AI and from actual AI use, neither of which was measured; the findings therefore concern appraisals and not AI adoption behavior. Such cognitive appraisal is associated not only with behavioral intention to adopt AI tools but, increasingly, with broader occupational adjustment outcomes (Venkatesh and Bala, 2008; Yim and Su, 2025).

From the perspective of Conservation of Resources theory, perceived AI acceptance constitutes an appraisal of a potential job resource: when PE teachers regard AI tools as useful and easy to operate, the technology is appraised as resource-conducive rather than resource-threatening, which is associated with reduced anticipatory resource loss and a greater sense of resource sufficiency (Hobfoll, 1989). Resource-sufficient individuals, in turn, tend to report more favorable affective experiences of their work, consistent with the Job Demands–Resources proposition that job and personal resources are linked to engagement and well-being (Bakker and Demerouti, 2017). Qualitative evidence among PE teachers further suggests that positive appraisals of AI’s instructional utility are associated with greater professional confidence, whereas ambivalent appraisals are linked to heightened occupational anxiety (Yang et al., 2026). Survey evidence among college PE teachers similarly indicates that AI usage intention is positively associated with digital competence, which can be viewed as a resource that may support smoother professional functioning (Yi et al., 2025). Direct evidence on PE teachers is limited, largely qualitative, or concerned with adjacent outcomes such as AI usage intention; evidence from general technology-acceptance and job-resource research is therefore used here as indirect, analogical support, and the hypotheses below should be read as theoretically derived propositions. Taken together, Hypothesis 1 (H1) is proposed: Perceived AI acceptance is positively associated with university PE teachers’ occupational well-being.

### 2.2 The mediating role of digital well-being

University PE teachers who appraise AI tools as useful and easy to use are more likely to integrate these tools into daily instructional, administrative, and research routines, which is associated with more frequent exposure to digital environments and their attendant self-regulatory demands (Gui et al., 2017). Digital well-being, conceptualized as the balance between individuals’ skills for managing digital information and communication flows and their subjective experience of digital-related stress, is conceptualized as a proximal personal resource that is related to both technological competence and the cognitive appraisal of technology (Gui et al., 2017). Within a COR framework, favorable perceptions of usefulness and ease of use are theoretically associated with a lower anticipated resource cost of digital engagement, which is consistent with teachers reporting effective digital self-regulation rather than digital overload (Han Z. et al., 2025).

Related empirical evidence is consistent with this reasoning, although it concerns neighboring constructs and not PE teachers’ digital well-being directly: workplace digitization has been shown to be associated with employees’ well-being partly through reduced digital anxiety, a construct closely aligned with the stress dimension of digital well-being (Han Z. et al., 2025). Digital well-being itself, reflecting a state of psychological and functional resource sufficiency in digital contexts, has been positively linked to broader occupational and subjective well-being outcomes, as individuals who feel less overwhelmed by digital demands retain more resources available for occupational engagement and positive affect (Diener et al., 1999; Gui et al., 2017). Accordingly, Hypothesis 2 (H2) is proposed: Perceived AI acceptance shows an indirect association with occupational well-being through digital well-being (statistical mediation).

### 2.3 The mediating role of innovative work behavior

Perceived AI acceptance is also associated with PE teachers’ innovative work behavior, defined as the intentional generation, promotion, and realization of novel ideas within one’s professional role (Janssen, 2000). When AI tools are appraised as useful and easy to operate, teachers are more likely to perceive them as resources that expand their repertoire of possible pedagogical solutions—for instance, AI-assisted movement analysis, personalized training-load monitoring, or automated instructional feedback (illustrative examples only, since this study measures general innovative work behavior in one’s work role and not teaching innovation specifically)— thereby being associated with greater idea generation (Cui et al., 2025; Yi et al., 2025). Consistent with this reasoning, recent organizational research indicates that generative AI use is positively associated with employees’ innovation performance through resource-based cognitive mechanisms, including resource acquisition and integration (Wang et al., 2025). This evidence comes from general organizational samples and not from teachers, and innovation performance is related to but not identical to innovative work behavior, so it is treated as indirect support.

From a COR perspective, perceived AI acceptance can be conceptualized as a resource-signaling appraisal: teachers who regard AI as usable and beneficial may be more willing to invest cognitive and behavioral resources in experimenting with novel approaches in their work, because the anticipated resource return may offset the resource investment required for innovation (Halbesleben et al., 2014; Hobfoll et al., 2018). Innovative work behavior, once enacted, is itself associated with heightened occupational well-being, as the successful promotion and realization of novel ideas is linked to experiences of competence and positive affect at work, consistent with a broader resource-gain spiral (Amabile, 1988; West and Farr, 1990); this evidence comes mainly from general innovation and creativity research and is used here as indirect support. Accordingly, Hypothesis 3 (H3) is proposed: Perceived AI acceptance shows an indirect association with occupational well-being through innovative work behavior (statistical mediation).

### 2.4 The serial mediating role of digital well-being and innovative work behavior

We propose that perceived AI acceptance may be positively associated with PE teachers’ occupational well-being through a serial pathway, sequentially involving digital well-being and innovative work behavior. This serial pathway is theoretically motivated by COR theory’s proposition of resource caravans, whereby resource gains tend to co-occur and build upon one another rather than operate in isolation (Halbesleben et al., 2014; Hobfoll et al., 2018). At the front end of this chain, perceived AI acceptance is associated with a more favorable appraisal of digital demands, which is consistent with a lower anticipated resource cost of technology engagement and with higher digital well-being as a personal resource (Gui et al., 2017).

Digital well-being, in turn, reflects not merely the absence of digital stress but also a surplus of regulatory and attentional capacity: teachers who are less burdened by digital overload may have more cognitive and motivational resources available for discretionary, proactive behaviors such as innovation (Ten Brummelhuis and Bakker, 2012; Han Z. et al., 2025). When digital well-being is high, teachers are more likely to possess the residual resource capacity to engage in the generation, promotion, and realization of novel ideas in their work role—that is, innovative work behavior—since innovation is itself a resource-intensive, discretionary behavior that draws on surplus cognitive and emotional resources (Bandura, 1986; Janssen, 2000). Finally, the successful enactment of innovative work behavior is associated with resource gains in the form of competence experiences, recognition, and positive work affect, which cumulatively are linked to occupational well-being (West and Farr, 1990; Spreitzer, 1995). Thus, the proposed sequence is one in which perceived AI acceptance is associated with digital well-being, digital well-being with innovative work behavior, and innovative work behavior with occupational well-being; this is a COR-consistent sequence proposed on theoretical grounds, and not a cascading process demonstrated by the data. Accordingly, Hypothesis 4 (H4) is proposed: Perceived AI acceptance shows a serial indirect association with university PE teachers’ occupational well-being through digital well-being and innovative work behavior in sequence (serial statistical mediation).

Why digital well-being is placed before innovative work behavior. Two considerations motivate this order. First, innovative work behavior is discretionary behavior that requires cognitive and motivational capacity; COR theory and the work-home resources literature suggest that individuals experiencing less strain are better positioned to invest in such behavior (Ten Brummelhuis and Bakker, 2012). Second, digital well-being, as conceptualized by Gui et al. (2017), is a state of skills and low stress in digital environments that is closely tied to technology use and appraisal, and it is therefore conceptually closer to the technological appraisal than innovative work behavior is. However, the reverse order is also theoretically plausible: innovative use of AI could build digital skills and reduce digital strain, and cross-sectional data cannot adjudicate between the two orders. The sequence tested here is thus a theoretically proposed order and not a temporally demonstrated one, and alternative orderings are examined in Section 4.7 and discussed in Section 5.6.

Based on the above hypotheses, the present study constructed a serial mediation model, as depicted in Figure 1, to examine whether the observed associations among these variables are consistent with the proposed sequence and to estimate the indirect associations of digital well-being and innovative work behavior (individually and in sequence) in the association between perceived AI acceptance and university PE teachers’ occupational well-being.

**Figure 1:**
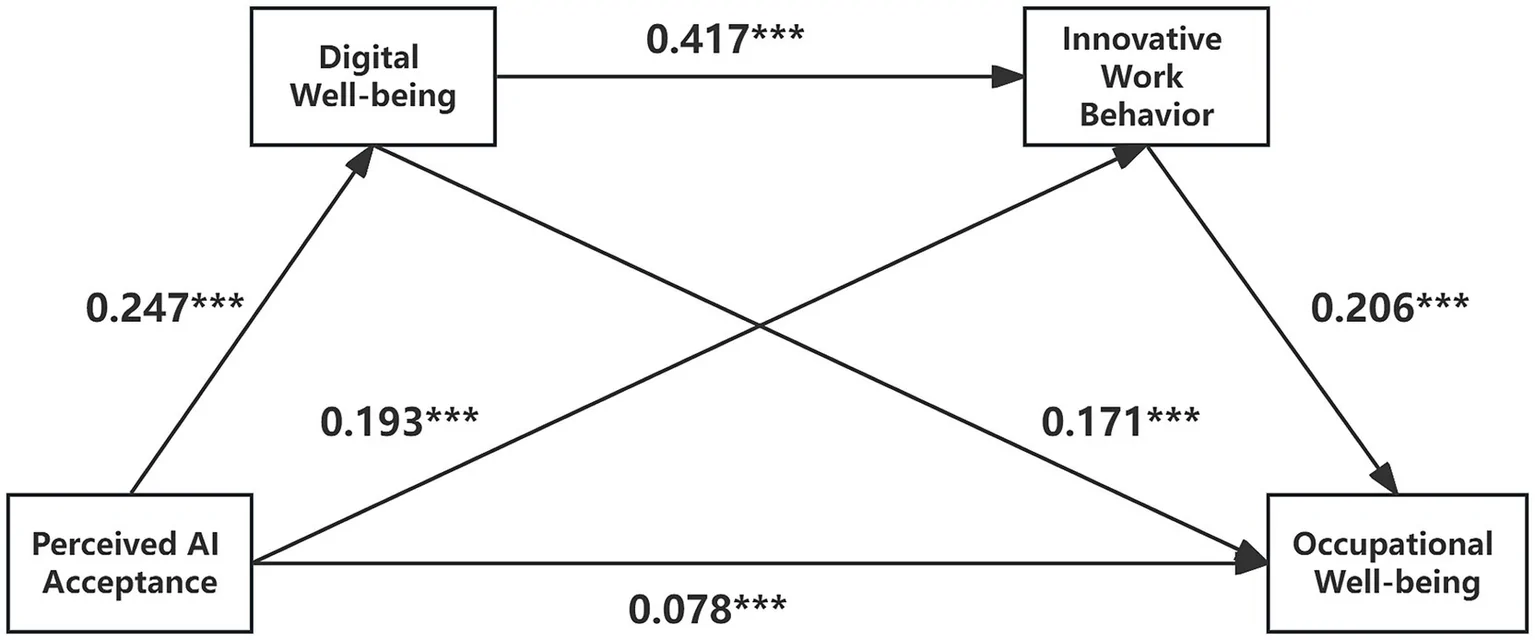
The hypothesized chain mediation model of perceived AI acceptance, digital well-being, innovative work behavior, and occupational well-being. ***p < 0.001.

## 3 Methods

### 3.1 Procedure and participants

This study employed a cross-sectional design. An anonymous electronic questionnaire was distributed to university physical education teachers via the Wenjuanxing online survey platform between 1 June 2026 and 10 August 2026. Participants were recruited through a combination of convenience sampling and snowball sampling, both non-probability methods: the questionnaire link was first sent to teachers who could be reached conveniently, who were asked to forward it to other university physical education teachers they knew. The questionnaire link was initially sent to teachers at 18 universities in six provinces and municipalities (Sichuan, Guangdong, Chongqing, Beijing, Hainan, and Hunan; the universities are listed in Supplementary Table S1) and was then circulated through snowball referral. Because the link was circulated through personal and professional networks, the number of invited teachers could not be determined, so a response rate cannot be calculated; the effective rate reported below is the proportion of returned questionnaires that were valid. Because of snowball referral, the universities that the questionnaire ultimately reached cannot be determined; the questionnaire was also anonymous and did not record respondent-level institutional affiliation, so the distribution of participants across universities cannot be reported. Gender, age, and years of teaching experience were also recorded, but because of problems in the collection and compilation of these data they could not be used, and none of the analyses reported here includes them. In total, 981 questionnaires were returned. The questionnaire consisted of four sections: the Perceived AI Acceptance Scale, the Digital Well-Being Scale, the Innovative Work Behavior Scale, and the Job-related Affective Well-being Scale (JAWS). Prior to participation, all respondents provided electronic informed consent, and all data were anonymized to ensure confidentiality. Data cleaning excluded 174 questionnaires in three mutually exclusive steps applied in the following order, so that no case was counted twice. The questionnaire contained, in addition to the four scales, a screening item on professional identity, an item on whether the respondent teaches university physical education classes and an item on weekly class hours, a pair of positively and reversely worded items on the use of a teaching method, and one attention-check item. First, 30 questionnaires were excluded because the screening item showed that the respondent was not a university physical education teacher (981–30 = 951 remaining). Second, of these 951, 113 were excluded because the total completion time was outside the limits set on the Wenjuanxing platform: a minimum of 1 min (60 s) and a maximum of 30 min (1,800 s). Wenjuanxing offers several completion-time limit options (for the maximum, for example, 5, 10, 15, and 20 min), these two limits were selected from them, and questionnaires outside the limits were marked as invalid by the platform and removed (951–113 = 838 remaining). The limits were platform settings and not empirically derived criteria. Third, of the remaining 838, 31 were excluded for internally inconsistent responses, defined as failing any one of the following checks: (a) answering “no” to whether the respondent teaches university physical education classes while reporting weekly class hours greater than 0; (b) answering “agree” or “strongly agree” to both the positively worded item “I often apply this teaching method in class” and the reversely worded item “I rarely apply this teaching method in class,” or, after reverse-scoring the negatively worded item, an absolute difference of 2 or more between the scores of the two items; and (c) not selecting the instructed option on the attention-check item (“Please select ‘somewhat agree’ for this item”). A total of 807 valid questionnaires (807/981 = 82.26%) were retained for analysis. The anonymized dataset will be retained in accordance with institutional data-management practice and made available to qualified researchers upon reasonable request, subject to a formal data-sharing agreement that protects participants’ privacy.

### 3.2 Ethical approval and informed consent

This study was conducted in accordance with the ethical principles of the Declaration of Helsinki. It did not involve invasive procedures, and all research methods complied with relevant guidelines and regulations. Ethical approval was obtained from the Biomedical Research Ethics Committee of Hunan Normal University (approval no. LSK2026-472) prior to the commencement of the study. The author, who is the project leader and the applicant, is affiliated with Sichuan Film and Television University; the ethical review was conducted by the Biomedical Research Ethics Committee of Hunan Normal University, which accepted the author’s entrustment to review the project (letter of acceptance of entrusted ethics review). The approved protocol specified online recruitment of university physical education teachers by convenience and snowball sampling in 18 universities in six provinces and municipalities (Sichuan, Guangdong, Chongqing, Beijing, Hainan, and Hunan) between 1 June and 10 August 2026; because of snowball sampling, the questionnaire reached universities across these provinces and municipalities. Data were collected anonymously and kept strictly confidential. All participants provided electronic informed consent and voluntarily agreed to participate; they retained the right to withdraw from the study at any time without penalty.

### 3.3 Measures

The Perceived AI Acceptance Scale was adapted from Davis (1989), with item wording modified to refer to AI tools used in university teaching. The Digital Well-Being Scale is a researcher-developed instrument: Gui et al. (2017) offer a theoretical framework and indicators of digital well-being but no ready-made item set, so the 12 items were compiled, translated, and reworded by the research team on the basis of that framework, and the scale should not be regarded as a previously validated instrument. The Innovative Work Behavior Scale and the JAWS were based on the original instruments (Janssen, 2000; Van Katwyk et al., 2000). The English items of the three published scales were translated into Chinese by one bilingual researcher, and a second bilingual expert who had not taken part in the translation checked the Chinese version against the original scales; disagreements were resolved through discussion in the research team. A pilot test with cognitive interviews was then conducted with 30 university physical education teachers, and the items were revised accordingly before the final version was set. Table 1 summarizes the source, the number of dimensions and items, and the Cronbach’s α of each scale.

**Table 1:** Scale sources and reliability coefficients.

Scale	Source	Dimensions	Items	Cronbach’s *α*
Perceived AI Acceptance (PAI/TAM)	Davis (1989)	2	12	0.897
Digital Well-Being (DWB)	Gui et al. (2017) (framework; researcher-developed items)	2	12	0.864
Innovative Work Behavior (IWB)	Janssen (2000)	3	9	0.830
Occupational Well-Being (JAWS)	Van Katwyk et al. (2000)	4	30	0.909

*N* = 807 for all scales, with no missing values.

#### 3.3.1 Perceived AI acceptance scale

Perceived AI acceptance was assessed using items adapted from the Technology Acceptance Model developed by Davis (Davis, 1989), comprising two dimensions: perceived usefulness (6 items, e.g., ‘Using this AI system would improve my job performance’) and perceived ease of use (6 items, e.g., ‘I would find the AI system easy to use’), for 12 items in total, treated as a single appraisal construct (see Section 2.1). Items were rated on a 7-point Likert-type scale (1 = strongly disagree, 7 = strongly agree), with higher scores indicating greater perceived AI acceptance. In the present study, the scale demonstrated a Cronbach’s α of 0.897.

#### 3.3.2 Digital well-being scale

Digital well-being was measured with a 12-item instrument developed by the research team on the basis of the theoretical framework and indicators proposed by Gui, Fasoli, and Carradore (Gui et al., 2017), comprising two dimensions: individual skills and behavior (6 items, e.g., ‘I can strategically filter and focus on valuable information channels and contacts’) and subjective perception and stress (6 items, reverse-scored, e.g., ‘I often feel that the time I spend online exceeds what I intended’), for 12 items in total. Items were rated on a 5-point scale (1 = strongly disagree, 5 = strongly agree), with higher scores after reverse-scoring indicating greater digital well-being. In this study, the scale showed a Cronbach’s α of 0.864. Because the instrument was developed for the present study, its psychometric evidence (Sections 4.3 and 4.7) is preliminary.

#### 3.3.3 Innovative work behavior scale

Innovative work behavior was measured using the nine-item scale of Janssen (2000), which is based on Scott and Bruce (1994), comprising three dimensions: idea generation (3 items, e.g., ‘creating new ideas for difficult issues’), idea promotion (3 items, e.g., ‘mobilizing support for innovative ideas’), and idea realization (3 items, e.g., ‘transforming innovative ideas into useful applications’), for 9 items in total. Respondents indicated how often they performed each behavior in the workplace on a 7-point scale (1 = never, 7 = always), with higher scores indicating more frequent innovative work behavior. This scale assesses general innovative work behavior in one’s work role and is not a measure of teaching or pedagogical innovation specifically. In the present study, the scale yielded a Cronbach’s α of 0.830.

#### 3.3.4 Occupational well-being scale

Occupational well-being was assessed using the Job-related Affective Well-being Scale (JAWS) developed by Van Katwyk, Fox, Spector, and Kelloway (Van Katwyk et al., 2000). The scale comprises 30 items describing job-related affective states along the pleasure and arousal axes (15 pleasurable and 15 displeasurable items), and assesses how frequently each state was experienced at work over the preceding 30 days; each item completes the stem “My job made me feel …” (e.g., “at ease,” “angry”). Following the original scoring logic, the displeasurable items were reverse-scored and all 30 items were averaged (rather than summed, so that scores remain on the 1–5 response metric) to form an overall occupational well-being score. Van Katwyk et al. (2000) also defined four subscales, each made up of the five most extreme items of one combination of pleasure and arousal: high-pleasure high-arousal (ecstatic, enthusiastic, excited, energetic, inspired), high-pleasure low-arousal (satisfied, content, at ease, relaxed, calm), low-pleasure high-arousal (furious, angry, frightened, anxious, disgusted), and low-pleasure low-arousal (depressed, discouraged, gloomy, fatigued, bored); the remaining 10 items contribute only to the overall score. In the present study, the four subscale scores were used as observed indicators of occupational well-being in the CFA, with the two low-pleasure subscales reverse-scored so that higher values indicate greater well-being. Items were rated on a 5-point scale (1 = never, 2 = rarely, 3 = sometimes, 4 = quite often, 5 = extremely often or always), with higher scores after reverse-scoring indicating greater occupational well-being. In this study, the overall 30-item scale demonstrated a Cronbach’s α of 0.909. The primary mediation analysis used this overall 30-item score, in line with the original scoring logic. The CFA, in contrast, used the four subscale means as indicators, so its indicators are based on 20 of the 30 items (the remaining 10 items contribute only to the overall score), and the CFA measurement model therefore does not map exactly onto the 30-item score used in the mediation analysis. This discrepancy is examined in a sensitivity analysis in which occupational well-being was computed from the 20 subscale items (Section 4.7).

### 3.4 Data analysis

The Kolmogorov–Smirnov test indicated that the distributions of perceived AI acceptance, digital well-being, and innovative work behavior departed significantly from normality (all *p* < 0.05); accordingly, Spearman’s rank-order correlation was used for all bivariate association analyses. Harman’s single-factor test was conducted as a preliminary check for potential common method bias; it is not regarded as conclusive evidence (see Section 4.2). The analysis was conducted in two separate steps and should not be read as a full covariance-based structural equation modeling (CB-SEM) analysis. In the first step, to evaluate the measurement structure underlying the four constructs, confirmatory factor analysis (CFA) was performed in AMOS 26.0 using maximum likelihood estimation (with bootstrap standard errors and bias-corrected 95% confidence intervals from 5,000 usable bootstrap samples used to address non-normality), with dimension-level mean scores (item parcels) as observed indicators of each latent construct (two indicators for perceived AI acceptance and digital well-being, three for innovative work behavior, and four for occupational well-being). These four JAWS indicators are based on 20 of the 30 JAWS items, whereas the occupational well-being score used in the second step is the mean of all 30 items (Section 3.3.4). Dimension-level parceling was adopted because the dimensions of each scale were specified *a priori*, because a 63-item model involves many parameters and is sensitive to minor item-level residual correlations and non-normality, and because the focus of the study was on the relations among constructs and not on item-level structure (Little et al., 2002). However, parceling can mask item-level multidimensionality and yield artificially favorable fit, particularly for adapted measures; the CFA is therefore interpreted as evidence on the dimension-level structure only, and an item-level CFA was added as a sensitivity analysis (Section 4.7). In the second step, the serial mediation model was tested using the PROCESS macro (version 5.0) in SPSS 27.0 with observed composite scores, with perceived AI acceptance as the independent variable, occupational well-being as the outcome variable, and digital well-being and innovative work behavior entered as sequential mediators (Model 6). Measurement and structural components were therefore not estimated jointly in a latent-variable framework. PROCESS Model 6 was chosen because it directly estimates serial multiple-mediator models and provides bootstrap confidence intervals for the specific and total indirect effects without assuming that indirect effects are normally distributed (Hayes, 2022). Because it relies on observed scores, measurement error is not modeled and the estimates may be attenuated relative to latent-variable estimates; full latent-variable SEM is recommended for future research (Section 5.6). The significance of indirect effects was determined using bootstrap resampling with 5,000 iterations and 95% bias-corrected confidence intervals; *p*-values are not reported for indirect effects. Because all variables were measured at the same time point, the indirect effects are interpreted as cross-sectional indirect associations and not as evidence of a temporal mediating process, which cross-sectional mediation estimates cannot establish (Maxwell and Cole, 2007). As robustness checks, alternative orderings of the two mediators were estimated, and the model was re-estimated with occupational well-being computed from the 20 items that define the four JAWS subscales (Section 4.7). Because participants were recruited from multiple universities by convenience and snowball sampling and respondent-level institutional affiliation was not recorded, possible clustering within universities could not be examined (Sections 4.7 and 5.6), and all analyses assume independent observations.

## 4 Results

### 4.1 Sample characteristics

After quality control, 807 valid questionnaires were analyzed (see Section 3.1 for the exclusion criteria and the number of cases excluded under each criterion). Of the 981 questionnaires returned, 174 were excluded (30 non-physical-education teachers, 113 with completion times outside the platform limits of 1 to 30 min, and 31 with inconsistent responses) and 807 (82.26%) were valid. The questionnaire was initially sent to teachers at 18 universities in six provinces and municipalities (Sichuan, Guangdong, Chongqing, Beijing, Hainan, and Hunan) and was then circulated through snowball referral. Because the questionnaire was anonymous, respondent-level institutional affiliation was not recorded, and because of problems in the collection and compilation of the gender, age, and teaching-experience data, these variables could not be used. No further sample profile can therefore be reported, and the distribution of participants across universities is unknown; this limits the assessment of representativeness (see Section 5.6).

### 4.2 Common method bias test

Because all data were collected through self-report at a single time point, the results might be susceptible to common method bias. Harman’s single-factor test was therefore conducted in SPSS 27.0 using an unrotated principal component analysis of all 63 items across the four scales. The first unrotated factor accounted for 19.365% of the total variance, below the 40% threshold. This result alone does not rule out common method variance, because Harman’s test has low sensitivity and does not statistically partial out method effects (Podsakoff et al., 2003); common method bias therefore cannot be excluded, and the associations reported below may be inflated or attenuated by shared method variance. More stringent procedures, such as an unmeasured latent method factor analysis or multi-source and multi-wave data, would be needed to examine this issue more rigorously.

### 4.3 Measurement model (confirmatory factor analysis)

To examine the structural validity of the four constructs, a four-factor measurement model (perceived AI acceptance, digital well-being, innovative work behavior, and occupational well-being) was tested in AMOS 26.0 using maximum likelihood estimation, with dimension-level mean scores used as observed indicators (item parcels); for occupational well-being, the four indicators were the means of the five items of each of the four JAWS subscales defined by Van Katwyk et al. (2000), with the two low-pleasure subscales reverse-scored. As shown in Table 2, the measurement model demonstrated good fit to the data: *χ*^2^(38) = 43.60, *p* = 0.246, *χ*^2^/df = 1.147, GFI = 0.990, AGFI = 0.983, NFI = 0.987, IFI = 0.998, TLI = 0.998, CFI = 0.998, RMSEA = 0.014 (90% CI [0.000, 0.029]), and SRMR = 0.018. All indices met or exceeded recommended thresholds (*χ*^2^/df < 3, CFI > 0.95, RMSEA < 0.08, SRMR < 0.08) (Hu and Bentler, 1999). Because parcels can yield more favorable fit than item-level models, these indices should be interpreted with caution (item-level results are reported in Section 4.7). The four indicators of occupational well-being are subscale means of the same instrument, and the subscales could not be distinguished in this sample (Section 4.7), so they reflect one overall construct; their high loadings (0.745–0.817) are therefore expected.

**Table 2:** Confirmatory factor analysis model fit indices.

Fit index	Value	Recommended standard
*χ* ^2^	43.596	–
df	38	–
*χ*^2^/df	1.147	<3
GFI	0.990	>0.90
AGFI	0.983	>0.90
NFI	0.987	>0.90
IFI	0.998	>0.90
TLI	0.998	>0.90
CFI	0.998	>0.95
RMSEA	0.014	<0.08
RMSEA 90% CI	[0.000, 0.029]	Upper bound < 0.08
SRMR	0.018	<0.08

As shown in Table 3, standardized factor loadings for all observed indicators ranged from 0.703 to 0.817 (*p* < 0.001), exceeding the 0.70 recommended threshold, and squared multiple correlations (*R*^2^) ranged from 0.494 to 0.667. As shown in Table 4, correlations among the four latent constructs ranged from 0.360 to 0.537, all below the 0.85 threshold (Kline, 1998). As shown in Table 5, CR and AVE were recalculated directly from the standardized CFA estimates in Table 3, using CR = (Σλ)^2^/[(Σλ)^2^ + Σ(1 − λ^2^)] and AVE = Σλ^2^/k, where λ is a standardized loading and k is the number of indicators of a construct. CR ranged from 0.725 to 0.870 and AVE ranged from 0.507 to 0.648, so that all CR values exceeded the 0.70 criterion and all AVE values exceeded the 0.50 criterion (Fornell and Larcker, 1981; Hair et al., 2010), supporting convergent validity at the dimension (parcel) level; the AVE of innovative work behavior (0.507) was only marginally above the criterion. In addition, the square root of each construct’s AVE (diagonal of Table 4: 0.805, 0.754, 0.712, and 0.791) exceeded the correlations of that construct with all other constructs (0.360 to 0.537), supporting discriminant validity according to the Fornell-Larcker criterion.

**Table 3:** Standardized factor loadings and squared multiple correlations (*R*^2^).

Latent variable	Observed indicator	Standardized loading	*R*^2^ (SMC)
PAI	Perceived Usefulness (PU)	0.801	0.642
	Perceived Ease of Use (PEOU)	0.809	0.654
DWB	Digital Skills	0.736	0.542
	Digital Stress (reversed)	0.772	0.596
IWB	Idea Generation	0.716	0.513
	Idea Promotion	0.703	0.494
	Idea Realization	0.718	0.516
OW (JAWS)	High-Pleasure High-Arousal	0.806	0.650
	High-Pleasure Low-Arousal	0.745	0.555
	Low-Pleasure High-Arousal (rev.)	0.817	0.667
	Low-Pleasure Low-Arousal (rev.)	0.794	0.630

All factor loadings were significant at *p* < 0.001. PAI = Perceived AI Acceptance; DWB = Digital Well-Being; IWB = Innovative Work Behavior; OW = Occupational Well-Being. Loadings are from the parcel-level CFA (dimension-level mean scores as indicators).

**Table 4:** Correlations among latent variables and discriminant validity.

Latent variable	1	2	3	4
1. PAI	**0.805**	–	–	–
2. DWB	0.490	**0.754**	–	–
3. IWB	0.403	0.495	**0.712**	–
4. OW	0.360	0.456	0.537	**0.791**

Off-diagonal entries are correlations among the latent constructs; diagonal entries (bold) are the square roots of the average variance extracted (AVE). Discriminant validity is supported when a diagonal value exceeds the correlations in the same row and column.

**Table 5:** Composite reliability (CR) and average variance extracted (AVE).

Latent variable	CR	AVE
PAI	0.786	0.648
DWB	0.725	0.569
IWB	0.756	0.507
OW	0.870	0.626

CR = composite reliability; AVE = average variance extracted. CR and AVE were calculated from the standardized loadings in Table 3 using CR = (Σλ)^2^/[(Σλ)^2^ + Σ(1 − λ^2^)] and AVE = Σλ^2^/k. All CR values exceeded the 0.70 recommended standard and all AVE values exceeded the 0.50 criterion (Fornell and Larcker, 1981; Hair et al., 2010).

### 4.4 Descriptive statistics and correlation analysis

Descriptive statistics and Spearman correlation coefficients among the observed study variables are presented in Table 6. Perceived AI acceptance was positively associated with digital well-being (*r* = 0.360, *p* < 0.001), innovative work behavior (*r* = 0.291, *p* < 0.001), and occupational well-being (*r* = 0.300, *p* < 0.001). Digital well-being was positively associated with innovative work behavior (*r* = 0.350, *p* < 0.001) and occupational well-being (*r* = 0.349, *p* < 0.001). Innovative work behavior was positively associated with occupational well-being (*r* = 0.440, *p* < 0.001). All variables were thus significantly and positively intercorrelated, indicating a pattern of associations that is consistent with, although it does not itself test, the proposed serial mediation model.

**Table 6:** Descriptive statistics and Spearman correlation analysis of variables.

Variables	*M*	SD	PAI	DWB	IWB	OW
Perceived AI Acceptance (PAI)	4.55	0.94	1			
Digital Well-being (DWB)	3.01	0.63	0.360**	1		
Innovative Work Behavior (IWB)	4.00	0.90	0.291**	0.350**	1	
Occupational Well-being (OW)	3.00	0.55	0.300**	0.349**	0.440**	1

*N* = 807. **p < 0.001 (two-tailed). Given significant departures from normality (Kolmogorov–Smirnov test, *p* < 0.05 for PAI, DWB, and IWB), Spearman’s rank-order correlation was used.

### 4.5 Chain mediation effect (primary analysis)

The serial mediation model (PROCESS Model 6), which constitutes the primary test of the hypothesized framework, was estimated with bootstrap resampling (5,000 iterations). As shown in Table 7, perceived AI acceptance was positively associated with digital well-being (a1 = 0.2468, SE = 0.0219, *t* = 11.258, *p* < 0.001) and innovative work behavior (a2 = 0.1933, SE = 0.0330, *t* = 5.853, *p* < 0.001); digital well-being was positively associated with innovative work behavior (*d* = 0.4168, SE = 0.0494, *t* = 8.444, *p* < 0.001); digital well-being was positively associated with occupational well-being (b1 = 0.1705, SE = 0.0297, *t* = 5.747, *p* < 0.001); and innovative work behavior was positively associated with occupational well-being (b2 = 0.2055, SE = 0.0203, *t* = 10.114, *p* < 0.001). The direct path from perceived AI acceptance to occupational well-being remained significant after accounting for both mediators (*c*′ = 0.0781, SE = 0.0194, *t* = 4.019, *p* < 0.001), and the total effect was *c* = 0.1810 (SE = 0.0196, *t* = 9.246, *p* < 0.001), supporting Hypothesis H1.

**Table 7:** Regression path coefficients of the chain mediation model (PROCESS Model 6).

Path	B (coeff.)	SE	*t*	*p*	95% CI
a1 (PAI → DWB)	0.2468	0.0219	11.258	<0.001	[0.2038, 0.2898]
a2 (PAI → IWB)	0.1933	0.0330	5.853	<0.001	[0.1285, 0.2581]
d (DWB → IWB)	0.4168	0.0494	8.444	<0.001	[0.3199, 0.5136]
b1 (DWB → OW)	0.1705	0.0297	5.747	<0.001	[0.1123, 0.2288]
b2 (IWB → OW)	0.2055	0.0203	10.114	<0.001	[0.1656, 0.2454]
c′ (PAI → OW, direct)	0.0781	0.0194	4.019	<0.001	[0.0399, 0.1162]
c (PAI → OW, total)	0.1810	0.0196	9.246	<0.001	[0.1426, 0.2195]

PAI = Perceived AI Acceptance; DWB = Digital Well-being; IWB = Innovative Work Behavior; OW = Occupational Well-being.

As shown in Table 8, the total indirect effect was 0.1030 (BootSE = 0.0116, 95% CI [0.0812, 0.1270]), accounting for 56.91% of the total effect, while the direct effect accounted for the remaining 43.09%. Among the three specific indirect pathways, the indirect effect through digital well-being alone (perceived AI acceptance → digital well-being → occupational well-being) was 0.0421 (BootSE = 0.0080, 95% CI [0.0271, 0.0586]), accounting for 23.26% of the total effect, consistent with Hypothesis H2. The indirect effect through innovative work behavior alone (perceived AI acceptance → innovative work behavior → occupational well-being) was 0.0397 (BootSE = 0.0076, 95% CI [0.0261, 0.0561]), accounting for 21.93% of the total effect, consistent with Hypothesis H3. The serial indirect effect through digital well-being and innovative work behavior in sequence (perceived AI acceptance → digital well-being → innovative work behavior → occupational well-being) was 0.0211 (BootSE = 0.0037, 95% CI [0.0149, 0.0297]), accounting for 11.66% of the total effect, consistent with Hypothesis H4. All bootstrap confidence intervals excluded zero, indicating statistically reliable indirect associations (inference is based on the bootstrap confidence intervals; *p*-values are not computed for indirect effects). The detailed pathways are illustrated in Figure 2.

**Table 8:** Indirect effects and bootstrap confidence intervals of the chain mediation model (PROCESS Model 6).

Effect	Path	Effect	BootSE	BootLLCI	BootULCI	Effect share
Ind1	PAI → DWB → OW	0.0421	0.0080	0.0271	0.0586	23.26%
Ind2	PAI → IWB → OW	0.0397	0.0076	0.0261	0.0561	21.93%
Ind3	PAI → DWB → IWB → OW	0.0211	0.0037	0.0149	0.0297	11.66%
Total indirect	Sum of Ind1–Ind3	0.1030	0.0116	0.0812	0.1270	56.91%
Direct effect	PAI → OW	0.0781	—	0.0399	0.1162	43.09%
Total effect	PAI → OW	0.1810	—	0.1426	0.2195	100%

Bootstrap resamples = 5,000; CI = bias-corrected percentile bootstrap confidence interval. Ind1: PAI → digital well-being → OW. Ind2: PAI → innovative work behavior → OW. Ind3: PAI → digital well-being → innovative work behavior → OW. PAI = Perceived AI Acceptance; DWB = Digital Well-being; IWB = Innovative Work Behavior; OW = Occupational Well-being. Inference for indirect effects is based on the bootstrap confidence intervals; *p*-values are not computed for indirect effects.

**Figure 2:**
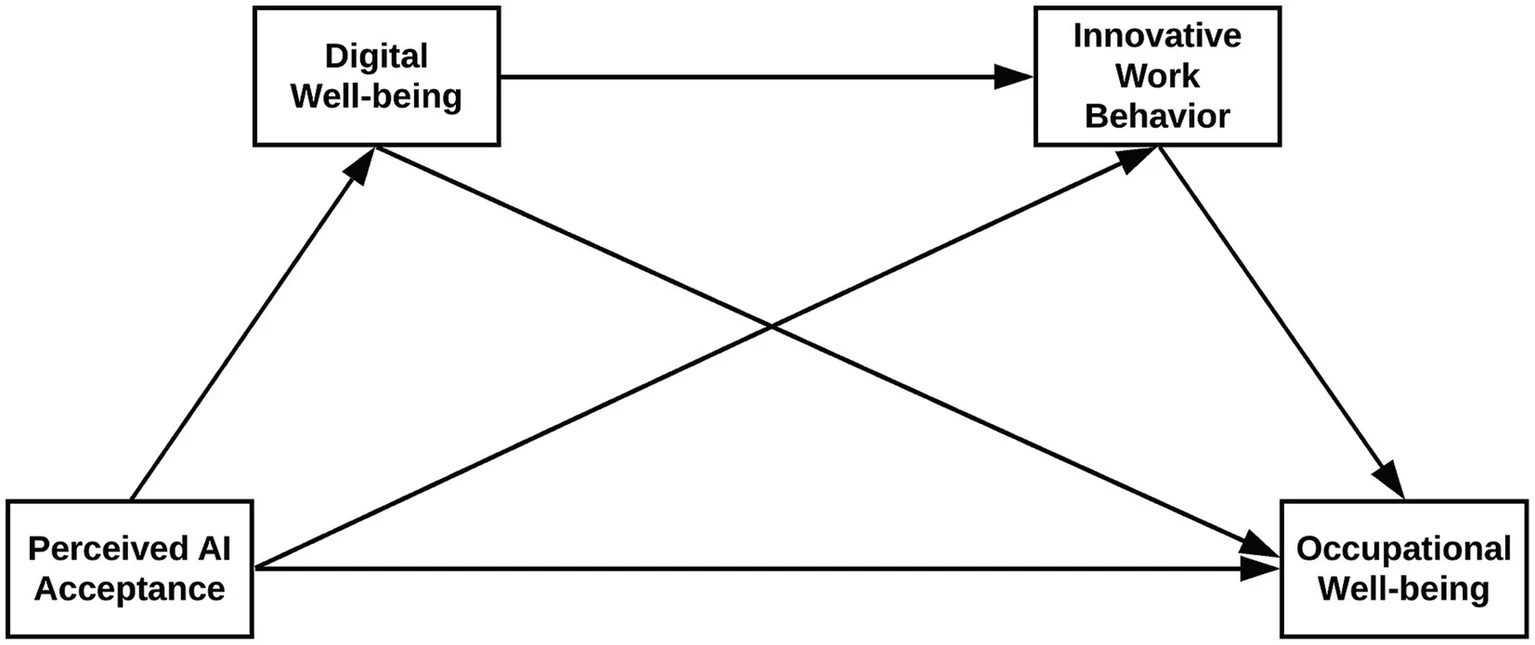
The chain mediation effect of digital well-being and innovative work behavior.

### 4.6 Supplementary analyses: simple mediation models

As supplementary analyses, simple mediation models (PROCESS Model 4) were estimated to examine each proposed mediator separately. Because each simple model omits the other mediator, its indirect effect is not directly comparable with the specific indirect effects of the serial model, which is the primary analysis (Section 4.5). As shown in Table 9, perceived AI acceptance was positively associated with digital well-being (*a* = 0.2468, SE = 0.0219, *t* = 11.258, *p* < 0.001), which in turn was positively associated with occupational well-being (*b* = 0.2562, SE = 0.0302, *t* = 8.490, *p* < 0.001); the direct path from perceived AI acceptance to occupational well-being remained significant (*c*′ = 0.1178, SE = 0.0202, *t* = 5.834, *p* < 0.001). The indirect effect via digital well-being alone was 0.0632 (BootSE = 0.0092, 95% CI [0.0468, 0.0829]), accounting for 34.92% of the total effect (*c* = 0.1810).

**Table 9:** Simple mediation models (PROCESS Model 4): path coefficients and bootstrap effect sizes (supplementary analysis).

Path/effect	*B* (coeff.)	SE	*t*	*p*	95% Bootstrap CI	Effect share
Model 4a: PAI → DWB → OW						
a (PAI → DWB)	0.2468	0.0219	11.258	<0.001	[0.2038, 0.2898]	–
b (DWB → OW)	0.2562	0.0302	8.490	<0.001	[0.1970, 0.3154]	–
c′ (PAI → OW, direct)	0.1178	0.0202	5.834	<0.001	[0.0782, 0.1574]	–
c (total effect)	0.1810	0.0196	9.246	<0.001	[0.1426, 0.2195]	100%
Indirect effect (a × b)	0.0632	BootSE = 0.0092	–	–	[0.0468, 0.0829]	34.92%
Model 4b: PAI → IWB → OW						
a (PAI → IWB)	0.2962	0.0320	9.252	<0.001	[0.2333, 0.3590]	–
b (IWB → OW)	0.2389	0.0199	12.027	<0.001	[0.1999, 0.2778]	–
c′ (PAI → OW, direct)	0.1103	0.0190	5.814	<0.001	[0.0731, 0.1475]	–
c (total effect)	0.1810	0.0196	9.246	<0.001	[0.1426, 0.2195]	100%
Indirect effect (a × b)	0.0707	BootSE = 0.0094	–	–	[0.0537, 0.0903]	39.06%

Bootstrap resamples = 5,000. CI = bias-corrected percentile bootstrap confidence interval. All path coefficients are unstandardized. PAI = Perceived AI Acceptance; DWB = Digital Well-being; IWB = Innovative Work Behavior; OW = Occupational Well-being. These simple mediation models are supplementary to the serial mediation model (Tables 7, 8).

Perceived AI acceptance also was positively associated with innovative work behavior (*a* = 0.2962, SE = 0.0320, *t* = 9.252, *p* < 0.001), which in turn was positively associated with occupational well-being (*b* = 0.2389, SE = 0.0199, *t* = 12.027, *p* < 0.001); the direct path remained significant (*c*′ = 0.1103, SE = 0.0190, *t* = 5.814, *p* < 0.001). The indirect effect via innovative work behavior alone was 0.0707 (BootSE = 0.0094, 95% CI [0.0537, 0.0903]), accounting for 39.06% of the total effect.

### 4.7 Robustness analyses

Three robustness analyses were conducted to evaluate the dependence of the findings on the proposed ordering, the parceling strategy, and the scoring of occupational well-being. First, because cross-sectional data cannot establish temporal order, PROCESS Model 6 was re-estimated with the mediators in reversed order (perceived AI acceptance → innovative work behavior → digital well-being → occupational well-being). The serial indirect effect in the reversed order was also statistically reliable (*B* = 0.0099, BootSE = 0.0023, 95% CI [0.0060, 0.0152]), compared with *B* = 0.0211 in the proposed order; the path from innovative work behavior to digital well-being was b = 0.1955 (SE = 0.0231, *p* < 0.001), and the total indirect effect was unchanged (0.1030, 95% CI [0.0812, 0.1263]). Because both orders yielded indirect effects whose confidence intervals excluded zero, the data cannot discriminate between them, and the proposed ordering rests on theoretical grounds only. Second, item-level CFAs were conducted for each scale separately using all 63 original items (AMOS 26.0, maximum likelihood estimation, items treated as continuous). When the items were specified on their *a priori* dimensions, fit was good for perceived AI acceptance [*χ*^2^(53) = 64.21, CFI = 0.997, TLI = 0.996, RMSEA = 0.016, SRMR = 0.020], digital well-being [*χ*^2^(53) = 68.02, CFI = 0.995, TLI = 0.993, RMSEA = 0.019, SRMR = 0.022], and innovative work behavior [*χ*^2^(24) = 30.23, CFI = 0.997, TLI = 0.995, RMSEA = 0.018, SRMR = 0.019]. The latent correlation between the two dimensions was 0.76 for perceived AI acceptance and 0.70 for digital well-being, and the correlations among the three innovative work behavior dimensions ranged from 0.69 to 0.74, all well below 0.85. One-factor item-level models for perceived AI acceptance (χ^2^(54) = 404.18, CFI = 0.908, TLI = 0.888, RMSEA = 0.090, 90% CI [0.082, 0.098], SRMR = 0.056) and digital well-being (*χ*^2^(54) = 376.95, CFI = 0.886, TLI = 0.860, RMSEA = 0.086, 90% CI [0.078, 0.094], SRMR = 0.060) fit significantly worse than the corresponding two-dimension models [Δ*χ*^2^(1) = 339.97 and 308.93, respectively, both *p* < 0.001; ΔCFI = 0.089 and 0.109], so the two dimensions of each construct are distinguishable at the item level. For JAWS, a single-factor model of all 30 items showed acceptable RMSEA and SRMR but a CFI below the conventional 0.90 level (*χ*^2^(405) = 1,062.57, CFI = 0.884, TLI = 0.876, RMSEA = 0.045, 90% CI [0.042, 0.048], SRMR = 0.042), with uniformly modest standardized loadings (0.44 to 0.57). When the 20 items that define the four JAWS subscales were modeled as four correlated factors, the solution was inadmissible [*χ*^2^(164) = 435.10, CFI = 0.918, TLI = 0.906, RMSEA = 0.045, SRMR = 0.040; latent correlations from 0.91 to 1.06, two of them above 1], whereas a single-factor model of the same 20 items fit almost identically [*χ*^2^(170) = 451.77, CFI = 0.915, TLI = 0.905, RMSEA = 0.045, SRMR = 0.040] and had the lower BIC (719.5 versus 743.0). The four JAWS subscales therefore could not be empirically distinguished in this sample, and occupational well-being was treated as a single overall score. Standardized item loadings ranged from 0.612 to 0.747 for perceived AI acceptance, 0.606 to 0.679 for digital well-being, 0.643 to 0.716 for innovative work behavior, and 0.440 to 0.566 for JAWS (all above 0.40). At the item level, composite reliability was acceptable (0.69 to 0.85 for the dimensions of the first three scales and 0.91 for JAWS), but average variance extracted was below 0.50 for every dimension (0.41 to 0.49) and for the single JAWS factor (0.25), so convergent validity is supported at the parcel level but not at the item level. The dimension-level structure of the first three scales was therefore largely replicated at the item level, although the item loadings, particularly those of JAWS, were modest; these item-level analyses are sensitivity analyses and do not remove the limitations of the parceled CFA. In addition, because respondent-level institutional affiliation was not recorded, dependence among teachers from the same university could not be examined; intraclass correlation coefficients and cluster-robust or multilevel estimates could not be computed, and the standard errors and bootstrap confidence intervals reported above assume independent observations (see Section 5.6).

Third, to examine whether the findings depend on how occupational well-being was scored, PROCESS Model 6 was re-estimated with occupational well-being computed as the mean of the 20 items that define the four JAWS subscales (the two low-pleasure subscales reverse-scored; Cronbach’s *α* = 0.871) in place of the 30-item mean used in the primary analysis. The 20-item score correlated *r* = 0.979 with the 30-item score (*M* = 2.98, SD = 0.57 versus *M* = 3.00, SD = 0.55). With identical settings (5,000 bias-corrected bootstrap samples), the estimates were virtually unchanged (Table 10): the total effect was *B* = 0.1805 (95% CI [0.1408, 0.2203]) and the direct effect was *B* = 0.0749 (95% CI [0.0353, 0.1146]); the total indirect effect was 0.1056 (BootSE = 0.0119, 95% CI [0.0835, 0.1303]); and the specific indirect effects were 0.0448 (BootSE = 0.0083, 95% CI [0.0295, 0.0622]) via digital well-being, 0.0397 (BootSE = 0.0077, 95% CI [0.0260, 0.0560]) via innovative work behavior, and 0.0211 (BootSE = 0.0037, 95% CI [0.0149, 0.0300]) through both in sequence. All bootstrap confidence intervals excluded zero, and the indirect effects accounted for 58.50% of the total effect (versus 56.91% with the 30-item score). The pattern of results therefore does not depend on whether occupational well-being is scored from all 30 items or from the 20 subscale items. Because the 20 items are a subset of the same instrument and share its method variance, this analysis addresses the scoring of the measure only and does not provide independent validation of it.

**Table 10:** Sensitivity analysis of the serial mediation model (PROCESS Model 6) with occupational well-being scored from 30 items and from 20 items.

Effect	30-item score, *B* [95% CI]	20-item score, *B* [95% CI]
Total effect (c)	0.1810 [0.1426, 0.2195]	0.1805 [0.1408, 0.2203]
Direct effect (c′)	0.0781 [0.0399, 0.1162]	0.0749 [0.0353, 0.1146]
DWB → OW (b1)	0.1705 [0.1123, 0.2288]	0.1814 [0.1209, 0.2419]
IWB → OW (b2)	0.2055 [0.1656, 0.2454]	0.2054 [0.1640, 0.2469]
Total indirect effect	0.1030 [0.0812, 0.1270]	0.1056 [0.0835, 0.1303]
Ind1: PAI → DWB → OW	0.0421 [0.0271, 0.0586]	0.0448 [0.0295, 0.0622]
Ind2: PAI → IWB → OW	0.0397 [0.0261, 0.0561]	0.0397 [0.0260, 0.0560]
Ind3: PAI → DWB → IWB → OW	0.0211 [0.0149, 0.0297]	0.0211 [0.0149, 0.0300]
Indirect effects as % of the total effect	56.91%	58.50%

*N* = 807. Coefficients are unstandardized. The 30-item score is the mean of all 30 JAWS items; the 20-item score is the mean of the 20 items that define the four JAWS subscales (the two low-pleasure subscales reverse-scored; Cronbach’s *α* = 0.871; *r* = 0.979 with the 30-item score). The paths from perceived AI acceptance to digital well-being (a1 = 0.2468) and to innovative work behavior (a2 = 0.1933) and from digital well-being to innovative work behavior (*d* = 0.4168) do not involve occupational well-being and are identical in both models. Confidence intervals for indirect effects are bias-corrected bootstrap intervals (5,000 resamples); intervals for path coefficients are conventional 95% confidence intervals. PAI = Perceived AI Acceptance; DWB = Digital Well-being; IWB = Innovative Work Behavior; OW = Occupational Well-being.

Because the digital well-being instrument was developed by the research team for this study and has no prior validation, its properties were examined in more detail. Item-level descriptive statistics showed that all 12 items had skewness between −0.20 and 0.11 and kurtosis between −0.56 and −0.36, with no missing values, and that no item had more than 13% of responses at either endpoint of the scale. Internal consistency was satisfactory for both dimensions (individual skills: α = 0.81, composite reliability = 0.81; subjective perception and stress: α = 0.81, composite reliability = 0.81; mean inter-item correlations = 0.42 and 0.41). Corrected item–total correlations ranged from 0.53 to 0.60, and deleting any item would have lowered α. Composite reliability was computed from the standardized loadings of the item-level CFA (0.62 to 0.68 and 0.61 to 0.67 for the two dimensions) and, because each item loads on one factor only, is equivalent to McDonald’s omega. Average variance extracted was 0.42 and 0.41, below 0.50, because the standardized loadings were moderate. These results support the reliability of the instrument but not strong item-level convergent validity, and its validity evidence should be regarded as preliminary.

## 5 Discussion

This study constructed and tested a serial mediation model examining whether perceived AI acceptance is associated with university PE teachers’ occupational well-being through the sequential mediating roles of digital well-being and innovative work behavior. The data were consistent with all four hypotheses, and the pattern of results is compatible with a resource-based interpretation of how favorable technological appraisal may be linked to occupational adaptation in a digitalizing higher-education context. Given the cross-sectional, single-source design, however, the results describe statistical indirect associations and not established psychological mechanisms or temporal processes.

### 5.1 The relationship between perceived AI acceptance and occupational well-being

This study found that perceived AI acceptance was significantly and positively associated with university PE teachers’ occupational well-being, supporting Hypothesis H1 and aligning with a growing body of evidence documenting positive associations between favorable technology appraisals and occupational adjustment among teachers (Yi et al., 2025; Yang et al., 2026). The association between perceived AI acceptance and occupational well-being was accompanied by indirect associations through both digital well-being and innovative work behavior, which were themselves intercorrelated. From the perspective of Conservation of Resources theory, perceived AI acceptance can be interpreted as a favorable appraisal of a potential job resource, which is consistent with lower anticipated resource loss and with higher levels of resources in digital and behavioral domains (Hobfoll, 1989; Hobfoll et al., 2018) (these resource states were not directly measured). Higher levels of such resources are, in turn, associated with more favorable affective experiences of work, consistent with the Job Demands–Resources proposition that job and personal resources are linked to engagement and well-being (Bakker and Demerouti, 2017).

Notably, similar to prior chain-mediation research on teacher occupational well-being (Nong et al., 2022), the serial indirect pathway identified in the present study, while statistically reliable, represented a modest proportion of the total effect (11.66%), indicating that the sequence from digital well-being to innovative work behavior accounts for only a modest part of the association between perceived AI acceptance and occupational well-being, and that other factors not measured here are likely to be involved. Given that prosocial and occupational outcomes are typically shaped by numerous factors beyond any single technological appraisal—such as institutional support, professional identity, and workload—the magnitude of the present associations is consistent with the broader literature’s understanding of teacher well-being as a multiply determined outcome, while still carrying meaningful implications for how institutions introduce and scaffold AI tools for PE faculty.

### 5.2 The mediating role of digital well-being

This study also found a statistically significant indirect association between perceived AI acceptance and university PE teachers’ occupational well-being through digital well-being, consistent with Hypothesis H2. This result indicates that perceived AI acceptance is not only directly associated with occupational well-being but also shows an indirect association with it through digital well-being (in a statistical sense). One possible interpretation, which these data cannot test directly, is that when AI tools are appraised as useful and easy to operate, teachers may be less likely to experience the technology as an additional demand and may be more likely to adopt selective, adaptive strategies for managing digital information and communication flows (Gui et al., 2017). Consistent with this reasoning, prior organizational research has shown that workplace digitization is associated with reduced digital anxiety, which in turn is linked to enhanced employee well-being (Han Z. et al., 2025). Within a COR framework, a favorable technological appraisal is theoretically linked to a lower anticipated resource cost of digital engagement, which may be related to higher digital well-being as a personal resource that is, in turn, associated with broader occupational well-being (Hobfoll, 1989; Diener et al., 1999). Thus, the emotional and cognitive experience of digital demands may be one relevant correlate linking PE teachers’ appraisal of AI and their overall occupational well-being.

### 5.3 The mediating role of innovative work behavior

The results showed that innovative work behavior had a significant indirect association linking perceived AI acceptance and university PE teachers’ occupational well-being, consistent with Hypothesis H3. This finding is compatible with a role for proactive, resource-investing behavior in linking technology appraisal and occupational adaptation, although the cross-sectional data do not establish this role. When PE teachers regard AI tools as beneficial and easy to use, they appear more willing to invest cognitive and behavioral resources in generating, promoting, and realizing novel ideas in their work, consistent with recent evidence that generative AI use is associated with employees’ innovation performance through resource acquisition and integration mechanisms (Wang et al., 2025). Innovative work behavior is, in turn, associated with occupational well-being, plausibly because the successful promotion and realization of novel ideas is linked to experiences of competence, recognition, and positive affect at work (West and Farr, 1990; Janssen, 2000). This finding is consistent with prior literature indicating that innovative behavior is associated with individual well-being outcomes (Amabile, 1988), and adds evidence on this association in the domain of AI-related technology appraisal among university PE teachers. Note that innovative work behavior was measured with Janssen’s general scale, which captures idea generation, promotion, and realization in one’s work role; it does not specifically capture teaching or pedagogical innovation. The instructional examples given above are therefore illustrations of how general innovative behavior might appear among PE teachers and not features of what was measured.

### 5.4 The serial mediating role of digital well-being and innovative work behavior

This study further found a statistically significant serial indirect association through digital well-being and innovative work behavior in sequence, consistent with Hypothesis H4. This pattern is compatible with a cumulative, resource-based sequence: from a favorable technological appraisal, to digital resource sufficiency, to proactive behavioral resource investment, and then to occupational well-being. Because all variables were measured at one time point, however, it should be understood as a statistical indirect association and not as evidence of a temporal cascade or a psychological mechanism.

From a Conservation of Resources perspective, resource gains are theorized to unfold as caravans or spirals in which one resource facilitates the acquisition of further resources (Hobfoll, 1989). Within this theoretical framework, perceived AI acceptance may be associated with reduced anticipated resource loss in digital contexts, and teachers may therefore be more likely to report digital well-being as a foundational personal resource. This surplus of digital-regulatory capacity may, in turn, be associated with a greater residual capacity for discretionary, resource-intensive behaviors such as innovation (Bandura, 1986; Ten Brummelhuis and Bakker, 2012). Innovative work behavior may then be linked to further resource gains—competence experiences and positive affect—that are associated with occupational well-being (West and Farr, 1990; Spreitzer, 1995). These statements describe the theoretical interpretation with which the observed associations are consistent; the present data do not show that one resource leads to another, and the same associations could arise from reversed or reciprocal relationships. Within the context of this study, perceived AI acceptance is therefore associated with occupational well-being both directly and indirectly through digital well-being and innovative work behavior, a pattern that is consistent with, but does not demonstrate, a COR-based resource sequence.

From a theoretical standpoint, this serial model brings two previously separate strands of literature—digital well-being research and innovative work behavior research—into a single analytic model and offers a COR-consistent interpretation of their joint associations with occupational well-being. Perceived AI acceptance is first associated with an internal, self-regulatory resource state (digital well-being), which is subsequently associated with an external, behaviorally enacted resource-investment process (innovative work behavior), and finally with occupational well-being. However, given the cross-sectional design of this study, this temporal ordering remains theoretical rather than established, and reciprocal or reverse associations (e.g., occupational well-being fostering more favorable appraisals of AI, or innovative work behavior contributing to digital well-being) cannot be ruled out. Future longitudinal or experimental research is needed to disentangle the directionality of these associations. The theoretical contribution to COR theory should also be stated cautiously. The present cross-sectional, single-source design primarily tests whether the observed associations are consistent with a COR-based conceptual model; its contribution lies in specifying distinct conceptual roles for appraisal, personal-condition resource, resource investment, and affective outcome, and in applying them to PE teachers’ AI acceptance. It does not test COR theory’s resource caravan or spiral propositions, which require longitudinal data, and it should not be read as an extension of the theory.

### 5.5 Practical implications for teacher education and professional development

The present findings suggest several possible implications for higher-education institutions seeking to support PE teachers’ occupational adaptation during AI integration. These suggestions are drawn from the observed cross-sectional associations; none of the interventions was tested in this study, and the findings do not demonstrate that they would improve occupational well-being. First, because perceived AI acceptance was associated with occupational well-being both directly and through digital well-being and innovative work behavior, institutions might consider exploring structured AI-literacy training aimed at accurate, confidence-building appraisals of AI tools’ usefulness and usability. Second, given the observed indirect association involving digital well-being, professional development programs might consider incorporating guidance on regulating digital workload and information demands, which could help prevent AI adoption from becoming an additional occupational stressor. Third, given the observed indirect association involving innovative work behavior, institutions might consider whether organizational conditions — such as protected time, peer exchange platforms, or recognition mechanisms — could allow PE teachers to put favorable technology appraisals into innovative work practices. Taken together, these possibilities suggest that supporting teachers’ psychological adaptation to AI may involve more than technology provision and may merit attention to teachers’ digital well-being and their opportunities for innovative work behavior; whether this is the case should be tested in longitudinal and intervention studies.

### 5.6 Limitations and future directions

Several limitations of the present study should be acknowledged. The most fundamental limitation is its cross-sectional design, with all variables measured at a single time point; accordingly, the possibility of reverse or reciprocal associations (e.g., occupational well-being shaping technology appraisal, or innovative work behavior shaping digital well-being) cannot be excluded. Future research is advised to adopt a multi-wave longitudinal design (e.g., T1: perceived AI acceptance; T2: digital well-being; T3: innovative work behavior and occupational well-being) with autoregressive effects controlled, in order to examine predictive ordering and potential reverse pathways more rigorously. Moreover, a reversed ordering of the mediators also yielded a statistically reliable serial indirect effect (Section 4.7), so the proposed ordering cannot be regarded as empirically established.

Second, all data were collected through self-report measures. Harman’s single-factor test is insufficient to rule out common method variance, which therefore cannot be excluded; self-report data may also be susceptible to social desirability and recall bias. Future studies could incorporate objective indicators of AI use (e.g., platform login and usage logs), peer or supervisor ratings of innovative work behavior, and physiological or behavioral indicators of digital stress, to triangulate findings and reduce mono-method bias.

Third, this study did not incorporate other potential confounders, such as institutional AI-training provision, disciplinary or sport-specialization differences, or organizational support for technology adoption, all of which could plausibly shape both perceived AI acceptance and its downstream associations. Future research should consider incorporating these variables, as well as testing potential moderators such as professional title, teaching experience, or institutional type, to provide a more comprehensive account of the proposed model. In addition, because of problems in the collection and compilation of the demographic data (gender, age, and years of teaching experience), these variables could not be used and were not controlled in any of the reported models, so the estimates may partly reflect demographic differences.

Fourth, the measurement model was evaluated by CFA in AMOS, whereas the serial mediation paths were estimated separately in PROCESS Model 6 using observed composite scores. The study therefore did not employ a full latent-variable structural equation model in which measurement and structural components are estimated jointly; measurement error is not modeled in the structural paths, which can bias the estimated indirect effects. In addition, the CFA relied on dimension-level parcels (only two indicators for perceived AI acceptance and digital well-being), which can mask item-level multidimensionality and favor model fit. Future research could employ a full latent-variable SEM with item-level indicators to jointly estimate measurement and structural components and to more comprehensively evaluate model fit. Item-level CFAs (Section 4.7) largely replicated the dimension-level structure, but the stand-alone JAWS model had a CFI of 0.884 and modest item loadings (0.440–0.566), so the measurement of occupational well-being merits refinement in future work. In addition, the CFA indicators for occupational well-being cover 20 of the 30 JAWS items, whereas the mediation analysis used the mean of all 30 items; a sensitivity analysis with the 20-item score yielded virtually identical estimates (Section 4.7), but both scores come from the same instrument and share its method variance.

Fifth, the evidence for convergent validity is bounded by the measurement design. Although the recalculated AVE values exceeded the 0.50 criterion, the AVE of innovative work behavior (0.507) was only marginally above it, each construct was indicated by only two to four dimension-level parcels, and one scale was adapted and one (digital well-being) was developed by the research team without prior validation. A substantial share of indicator variance (e.g., 1 − AVE = 0.49 for innovative work behavior) is therefore still attributable to error and unique variance, which may attenuate the estimated associations. Future research could refine the items, for example by shortening and clarifying the adapted scales, testing item-level models, and cross-validating the scales in independent samples, to enhance convergent validity. In addition, the four JAWS subscales could not be empirically distinguished in this sample (latent correlations of 0.91 to 1.06 in the item-level model), so the occupational well-being parcels should be read as indicators of a single overall construct and not as evidence for four separable dimensions.

Sixth, perceived AI acceptance combined perceived usefulness and ease of use into a single appraisal construct, so their separate associations were not examined, and innovative work behavior was measured with a general scale and not with a specific measure of teaching innovation; conclusions about teaching or pedagogical innovation should therefore not be drawn. In addition, the sample was obtained through non-probability convenience and snowball sampling at multiple universities in six provinces and municipalities, so it may not be representative of university PE teachers in China, and self-selection and network homophily cannot be excluded. Moreover, because institutional affiliation was not recorded and the gender, age, and teaching-experience data could not be used, the sample cannot be described demographically, the estimates could not be adjusted for these variables, and possible clustering of observations within universities could not be examined; if teachers from the same university resemble one another, the reported standard errors and confidence intervals may be too narrow.

Finally, the present sample was drawn from university PE teachers in China; caution should therefore be exercised in generalizing these findings to other teacher populations, disciplinary contexts, or cultural settings. Cross-cultural and cross-disciplinary replication would help clarify the boundary conditions of the proposed model.

## 6 Conclusion

Based on Conservation of Resources theory, this cross-sectional study examined whether a serial mediation model of ‘perceived AI acceptance → digital well-being → innovative work behavior → occupational well-being’ was consistent with data from university PE teachers, and drew the following main conclusions.

First, perceived AI acceptance was significantly and positively associated with university PE teachers’ occupational well-being (total effect *B* = 0.181), and a direct association (*B* = 0.078) remained after the two mediators were accounted for.

Second, statistically significant indirect associations were observed between perceived AI acceptance and occupational well-being through digital well-being (*B* = 0.042; 23.26% of the total effect), through innovative work behavior (*B* = 0.040; 21.93%), and through both in sequence (*B* = 0.021; 11.66%), while the direct association accounted for 43.09%. This pattern is consistent with the proposed serial model. However, because the data are cross-sectional and self-reported, the proposed ordering is a theoretical proposition and not an established temporal mechanism, and alternative orderings cannot be excluded.

Third, these findings may be of interest to institutions seeking to support PE teachers’ occupational well-being during AI integration, but any practical implications are tentative and should be tested in longitudinal and intervention studies.

## Data Availability

The original contributions presented in the study are included in the article/Supplementary material, further inquiries can be directed to the corresponding author.

## References

[ref1] Amabile T. M. (1988). A model of creativity and innovation in organizations. Res. Organ. Behav. 10, 123–167.

[ref2] Bakker A. B. Demerouti E. (2017). Job demands–resources theory: taking stock and looking forward. J. Occup. Health Psychol. 22, 273–285. doi: 10.1037/ocp0000056, 27732008

[ref3] Bandura A. (1986). Social Foundations of Thought and Action. A Social Cognitive Theory. Englewood Cliffs NJ: Prentice-Hall, 2.

[ref4] Cui B. Jiao W. Gui S. Li Y. Fang Q. (2025). Innovating physical education with artificial intelligence: a potential approach. Front. Psychol. 16:1490966. doi: 10.3389/fpsyg.2025.1490966, 40276666 PMC12018358

[ref5] Davis F. D. (1989). Perceived usefulness, perceived ease of use, and user acceptance of information technology. MIS Q. 13, 319–340. doi: 10.2307/249008

[ref6] Diener E. Suh E. M. Lucas R. E. Smith H. L. (1999). Subjective well-being: three decades of progress. Psychol. Bull. 125, 276–302. doi: 10.1037/0033-2909.125.2.276

[ref7] Fornell C. Larcker D. F. (1981). Evaluating structural equation models with unobservable variables and measurement error. J. Mark. Res. 18, 39–50. doi: 10.1177/002224378101800104

[ref8] Gui M. Fasoli M. Carradore R. (2017). “Digital well-being”. Developing a new theoretical tool for media literacy research. Ital. J. Sociol. Educ. 9, 155–173. doi: 10.14658/pupj-ijse-2017-1-8

[ref9] Hair J. F. Black W. C. Babin B. J. Anderson R. E. (2010). Canonical correlation: a supplement to multivariate data analysis. 7th ed (Up. Saddle River NJ USA: Pearson Prentice Hall).

[ref10] Halbesleben J. R. B. Neveu J.-P. Paustian-Underdahl S. C. Westman M. (2014). Getting to the “COR”: understanding the role of resources in conservation of resources theory. J. Manag. 40, 1334–1364. doi: 10.1177/0149206314527130

[ref11] Han X. Chen F. Wang H. Xu S. (2025). Unlocking innovation: artificial intelligence usage and innovative behavior in the workplace. Soc. Behav. Pers. 53, 1–13. doi: 10.2224/sbp.13851

[ref12] Han Z. Song G. Zhang Y. Yan L. (2025). Enhancing employees’ workplace well-being through workplace digitization: exploring the mediating roles of job demands and digital anxiety. Front. Psychol. 16:1642489. doi: 10.3389/fpsyg.2025.1642489, 40787118 PMC12333450

[ref13] Hayes A. F. (2022). Introduction to Mediation, Moderation, and Conditional Process Analysis: A Regression-Based Approach. 3rd Edn New York, NY: Guilford Press.

[ref14] Hobfoll S. E. (1989). Conservation of resources: a new attempt at conceptualizing stress. Am. Psychol. 44, 513–524. doi: 10.1037/0003-066X.44.3.513, 2648906

[ref15] Hobfoll S. E. Halbesleben J. Neveu J.-P. Westman M. (2018). Conservation of resources in the organizational context: the reality of resources and their consequences. Annu. Rev. Organ. Psychol. Organ. Behav. 5, 103–128. doi: 10.1146/annurev-orgpsych-032117-104640

[ref16] Hu L. Bentler P. M. (1999). Cutoff criteria for fit indexes in covariance structure analysis: conventional criteria versus new alternatives. Struct. Equ. Model. 6, 1–55. doi: 10.1080/10705519909540118

[ref17] Janssen O. (2000). Job demands, perceptions of effort-reward fairness and innovative work behaviour. J. Occup. Organ. Psychol. 73, 287–302. doi: 10.1348/096317900167038

[ref18] Kline R. B. (1998). Structural Equation Modeling, vol. 33 New York: Guilford.

[ref19] Little T. D. Cunningham W. A. Shahar G. Widaman K. F. (2002). To parcel or not to parcel: exploring the question, weighing the merits. Struct. Equ. Model. 9, 151–173. doi: 10.1207/s15328007sem0902_1

[ref20] Maxwell S. E. Cole D. A. (2007). Bias in cross-sectional analyses of longitudinal mediation. Psychol. Methods 12, 23–44. doi: 10.1037/1082-989X.12.1.23, 17402810

[ref21] Nong L. Wu Y.-F. Ye J.-H. Liao C. Wei C. (2022). The effect of leisure engagement on preschool teachers’ job stress and sustainable well-being. Front. Psychol. 13:912275. doi: 10.3389/fpsyg.2022.912275, 35936277 PMC9353011

[ref22] Podsakoff P. M. MacKenzie S. B. Lee J.-Y. Podsakoff N. P. (2003). Common method biases in behavioral research: a critical review of the literature and recommended remedies. J. Appl. Psychol. 88, 879–903. doi: 10.1037/0021-9010.88.5.879, 14516251

[ref23] Scott S. G. Bruce R. A. (1994). Determinants of innovative behavior: a path model of individual innovation in the workplace. Acad. Manag. J. 37, 580–607. doi: 10.2307/256701

[ref24] Spreitzer G. M. (1995). Psychological, empowerment in the workplace: dimensions, measurement and validation. Acad. Manag. J. 38, 1442–1465. doi: 10.2307/256865

[ref25] Ten Brummelhuis L. L. Bakker A. B. (2012). A resource perspective on the work–home interface: the work–home resources model. Am. Psychol. 67, 545–556. doi: 10.1037/a0027974, 22506688

[ref26] Teng D. Shen Y. Yuan R. Wang W. (2026). Generative AI usage and employee innovation performance: the chain mediation mechanism of resource acquisition, integration, and activation. Front. Psychol. 17:1923950. doi: 10.3389/fpsyg.2026.1923950, 42769067 PMC13590364

[ref27] Van Katwyk P. T. Fox S. Spector P. E. Kelloway E. K. (2000). Using the job-related affective well-being scale (JAWS) to investigate affective responses to work stressors. J. Occup. Health Psychol. 5, 219–230. doi: 10.1037/1076-8998.5.2.219, 10784286

[ref28] Venkatesh V. Bala H. (2008). Technology acceptance model 3 and a research agenda on interventions. Decis. Sci. 39, 273–315. doi: 10.1111/j.1540-5915.2008.00192.x

[ref29] Venkatesh V. Morris M. G. Davis G. B. Davis F. D. (2003). User acceptance of information technology: toward a unified view1. MIS Q. 27, 425–478. doi: 10.2307/30036540

[ref30] Wang L. Qiao T. Wang X. Wang C. Ye P. (2025). The impact of work–family conflict on early childhood teachers’ occupational well-being: the chain mediating role of psychological empowerment and job crafting. Front. Public Health 12:1513514. doi: 10.3389/fpubh.2024.1513514, 39845653 PMC11753349

[ref31] Warr P. (1990). The measurement of well-being and other aspects of mental health. J. Occup. Psychol. 63, 193–210. doi: 10.1111/j.2044-8325.1990.tb00521.x

[ref32] West M. A. Farr J. L. (1990). Innovation and Creativity at Work: Psychological and Organizational Strategies. Chichester, UK: John Wiley & Sons.

[ref33] Yang W. Chen H. Rao F. (2026). A qualitative study of physical education teachers’ perceptions of artificial intelligence and influencing factors based on social cognitive theory. BMC Psychol. 14:530. doi: 10.1186/s40359-026-04295-x, 41792799 PMC13081590

[ref34] Yi R. Liu D. Sun X. Zhou B. (2025). Exploring the link between AI usage intention and digital competence among college PE teachers: a moderated mediation model based on SCT and UTAUT. PLoS One 20:e0334699. doi: 10.1371/journal.pone.0334699, 41270102 PMC12637994

[ref35] Yim I. H. Y. Su J. (2025). Artificial intelligence (AI) learning tools in K-12 education: a scoping review. J. Comput. Educ. 12, 93–131. doi: 10.1007/s40692-023-00304-9

[ref36] Zhang C. Schießl J. Plößl L. Hofmann F. Gläser-Zikuda M. (2023). Acceptance of artificial intelligence among pre-service teachers: a multigroup analysis. Int. J. Educ. Technol. High. Educ. 20:49. doi: 10.1186/s41239-023-00420-7

