# Supplementary material for: Perceived AI acceptance and occupational well-being among university physical education teachers: a cross-sectional serial mediation model of digital well-being and innovative work behavior
Source: Front Psychol. 2026 Oct 26;17:1999813. doi: 10.3389/fpsyg.2026.1999813 (PMC13745456; doi:10.3389/fpsyg.2026.1999813)
Supplement: Supplementary file 1 [file Table_1.docx]

**Supplementary Table S1**

*Universities to which the questionnaire link was initially sent (18 universities in six provinces and municipalities)*

| **No.** | **Province / municipality** | **University** | **Chinese name** |
| --- | --- | --- | --- |
| 1 | Sichuan | Sichuan Film and Television University | 四川电影电视学院 |
| 2 | Sichuan | Chengdu Sport University | 成都体育学院 |
| 3 | Sichuan | Sichuan Normal University | 四川师范大学 |
| 4 | Sichuan | Sichuan University | 四川大学 |
| 5 | Sichuan | University of Electronic Science and Technology of China | 电子科技大学 |
| 6 | Sichuan | Chengdu University | 成都大学 |
| 7 | Chongqing | Chongqing Second Normal University | 重庆第二师范学院 |
| 8 | Chongqing | Chongqing University of Arts and Sciences | 重庆文理学院 |
| 9 | Chongqing | Chongqing University | 重庆大学 |
| 10 | Hunan | Hunan Normal University | 湖南师范大学 |
| 11 | Hunan | Hunan University of Chinese Medicine | 湖南中医药大学 |
| 12 | Hunan | Hunan University of Technology and Business | 湖南工商大学 |
| 13 | Beijing | Capital University of Physical Education and Sports | 首都体育学院 |
| 14 | Beijing | Beijing Normal University | 北京师范大学 |
| 15 | Hainan | Hainan Normal University | 海南师范大学 |
| 16 | Hainan | Qiongtai Normal University | 琼台师范学院 |
| 17 | Guangdong | Jinan University | 暨南大学 |
| 18 | Guangdong | Guangzhou Sport University | 广州体育学院 |

*Note: The questionnaire was then circulated through snowball referral; the universities that it ultimately reached, and the distribution of respondents across universities, cannot be determined because respondent-level institutional affiliation was not recorded.*
